# Ex-PRESS Implantation Versus Trabeculectomy in Uncontrolled Glaucoma: A Meta-Analysis

**DOI:** 10.1371/journal.pone.0063591

**Published:** 2013-05-31

**Authors:** Wei Wang, Minwen Zhou, Wenbin Huang, Xiulan Zhang

**Affiliations:** 1 Zhongshan Ophthalmic Center, State Key Laboratory of Ophthalmology, Sun Yat-Sen University, Guangzhou, People's Republic of China; Zhongshan Ophthalmic Center, China

## Abstract

**Objective:**

To evaluate the efficacy and tolerability of Ex-PRESS implantation (Ex-Press) compared with trabeculectomy (Trab) in the treatment of patients with uncontrolled glaucoma.

**Methods:**

A comprehensive literature meta-analysis was performed according to the Cochrane Collaboration methodology to identify controlled clinical trials comparing Ex-Press with Trab. Efficacy estimates were measured by weight mean difference (WMD) for the percentage intraocular pressure (IOP) reduction from baseline to end-point, odds ratio (OR) for complete success, and qualified success rates. Tolerability estimates were measured by OR for adverse events. All outcomes were reported with a 95% confidence interval (CI). Data were synthesized by Stata 11.0 SE for Windows.

**Results:**

Eight controlled clinical trials meeting the predefined criteria were included in the meta-analysis. A total of 605 eyes from 559 patients with medically uncontrolled glaucoma were included. The weighted mean difference of the percentage IOP reduction from baseline was 2.33 (95% confidence interval: −2.59–7.24) when comparing Ex-Press with Trab. Ex-Press was associated with numerically greater, but nonsignificant, IOP lowering efficacy than Trab. The pooled odds ratio comparing Ex-Press with Trab were 0.93 (0.39, 2.23) for the complete success rate and 1.00 (0.39, 2.56) for the qualified success rate. Ex-Press was associated with a significantly lower frequency of hypotony and hyphema than Trab, with pooled ORs of 0.29 (0.13, 0.65) and 0.36 (0.13, 0.97), respectively.

**Conclusion:**

Ex-Press was associated with equivalent efficacy to Trab in lowering IOP. Comparable proportions of patients reached the IOP target with Ex-Press and Trab. Ex-Press was better tolerated than Trab.

## Introduction

Glaucoma is a leading cause of blindness. It has been estimated that over 11.1 million people world-wide will be bilaterally blind from primary glaucoma by 2020 [1]. Glaucoma treatments are directed at reducing intraocular pressure (IOP), either pharmacologically or surgically. Surgery is performed when medication and laser treatment cease to control IOP.

Trabeculectomy (Trab) has been the standard IOP-lowering procedure since the 1970s [2]. However, this technique is still associated with a significant rate of postoperative complications, including early hypotony, choroidal detachment, hypotonic maculopathy, endophthalmitis, along with others, which has prompted calls for a better and safer operation.

Ex-PRESS (Ex-Press), which is a small, stainless steel, non-valved shunt, is used in one of the most recently developed antiglaucomatous filtration surgical techniques. Currently, four types of Ex-PRESS (R-50, X-model, P-50, and P-200) are available [3]. Ex-Press was originally intended for direct implantation through the sclera under the conjunctiva, but this approach suffered from high rates of complication, including conjunctival erosion. Ex-Press has subsequently been modified to be placed under a scleral ﬂap, making it similar to the traditional Trab, while avoiding the need for scleral removal and iridectomy [4]. The theoretical advantage of this approach was increased reproducibility, simplicity, and reduced possibility of trauma to the ocular tissue. Over the last decade, it has been used successfully in approximately 60,000 implantations worldwide [5].

Several studies have compared Ex-Press with Trab. Some of the studies found that the Ex-Press group produced better results [6], [7], while another study found comparable results between the two groups [8]. These inconsistent results make it difficult to draw conclusions that could be applied in clinical practice. To our knowledge, the data have not been systematically evaluated and reported. Therefore, to assess the efficacy and tolerability of these two surgical procedures for the management of uncontrolled glaucoma, we undertook a meta-analysis of all available controlled clinical trials.

## Materials and Methods

This meta-analysis was performed according to a predetermined protocol (described in the following paragraph). Additionally, the standard systematic review guidelines, as outlined by the Cochrane Handbook for Systematic Reviews of Interventions [9], were followed at all stages of the process.

### 1. Literature search

Five electronic databases – PubMed, ISI Web of Science, EMBASE, the Chinese Biomedicine Database, and the Cochrane Library – were searched systematically for studies published before December 2012. The following terms, adapted for each database, were used for the searches: *Ex-PRESS*, *glaucoma*, and *trabeculectomy*. The Internet was searched using the Google search engine. A manual search was performed by checking the reference lists of original reports and review articles, retrieved through electronic searches, to identify studies not yet included in the computerized databases. The final search was carried out on December 2012, without restrictions regarding publication year or language.

### 2. Inclusion and exclusion criteria

The articles were considered eligible if the studies met the following inclusion criteria: (i) study type: comparative studies; (ii) population: glaucoma patients (but not including normal-tension glaucoma or ocular hypertension) fail to conservative therapy; (iii) intervention: Ex-Press versus Trab; (iv) outcome variables: at least one of the outcomes of interest was included. Abstracts from conferences and full texts without raw data available for retrieval, duplicate publications, letters, and reviews were excluded. For publications reporting on the same study population, the article reporting the results of the last end-point was included, and data that could not be obtained from this publication were obtained from others.

### 3. Outcome Measures

For efficacy, the primary outcome was the percentage of the IOP reduction (IOPR%). When authors reported the mean and standard deviation (SD) of the IOPR%, we used these directly. For studies that only reported absolute values for the IOP at baseline and end-point, the IOP reduction (IOPR) and the SD of the IOPR (SD_IOPR_) were calculated as follows: IOPR  =  IOP_baseline_ – IOP_end-point_, SD_IOPR_  =  (SD_baseline_^2^ + SD_end-point_^2^ – SD_baseline_ × SD_end-point_)^1/2^, then the IOPR% and the SD of the IOPR% (SD_IOPR%_) were estimated by IOPR%  =  IOPR/IOP_baseline,_ SD_IOPR%_  =  SD_IOPR_/IOP_baseline_.

The secondary outcome measure was the proportion of patients with *complete success*, which was defined as target end-point IOP without antiglaucoma medication; and *qualified success*, defined as target end-point IOP with or without antiglaucoma medications. We assessed tolerability by considering the proportions of patients with postoperative complications, including hypotony, choroidal effusion, flat anterior chamber, hypotony maculopathy, hyphema, bleb leak, and endophthalmitis.

### 4. Data extraction

The data were extracted independently by two reviewers (W.W. and Z.M.W.) and were rechecked after the first extraction. Disagreements were resolved through discussion. The information extracted from each study included the authors of each study, the year of publication, information on study design, location of the trial, duration of the study, number of subjects, age, sex, IOP measurements, and success rate. The numbers of withdrawals and patients reporting adverse events were also recorded.

### 5. Assessment of methodology quality

The qualities of clinical trials included were assessed by two independent observers (W.W. and Z.M.W.) using a system that can assess both randomized and non-randomized studies. This system was previously reported on by Downs and Blacks [10]. The system comprises 27 items distributed between 5 subscales regarding reporting (10 items), external validity (3 items), bias (7 items), confounding (6 items), and power (1 item). Any discrepancy in the qualitative assessment between the two observers was discussed and a consensus was reached. The total score of each trial was expressed as a percentage of the maximum achievable score. The studies with a quality score ≥50% were considered to have adequate quality.

### 6. Statistical analysis

The outcome measures were assessed on an ITT basis. Given that some of the trials did not report all the outcomes of interest, for each comparison and outcome, we did separate meta-analyses. Considering the differences in clinical characteristics among study groups and the variation in sample size, it was assumed that heterogeneity was present even when no statistical significance was identified, and it was decided to combine data using a random-effects model. For continuous variables, the weighted mean difference (WMD) was measured, while the odds ratios (OR) were measured for dichotomous variables. Both outcomes were reported with a 95% confidence interval (CI). Statistical heterogeneity among studies was evaluated with the χ2 and I^2^ tests. P<0.05 was considered statistically significant on the test for overall effect. Analysis was conducted using the Stata software package (Version 11.0; Stata Corp., College Station, TX).

### 7. Sensitivity analysis and publication bias

Sensitivity analysis was undertaken to evaluate the effect of methodological characteristics of controlled clinical trials in terms of trial design, which was differentiated as retrospective, prospective non-randomized, and randomized. To detect publication biases, we calculated Begg and Egger measures.

## Results

### 1. Literature search

**Figure 1:**
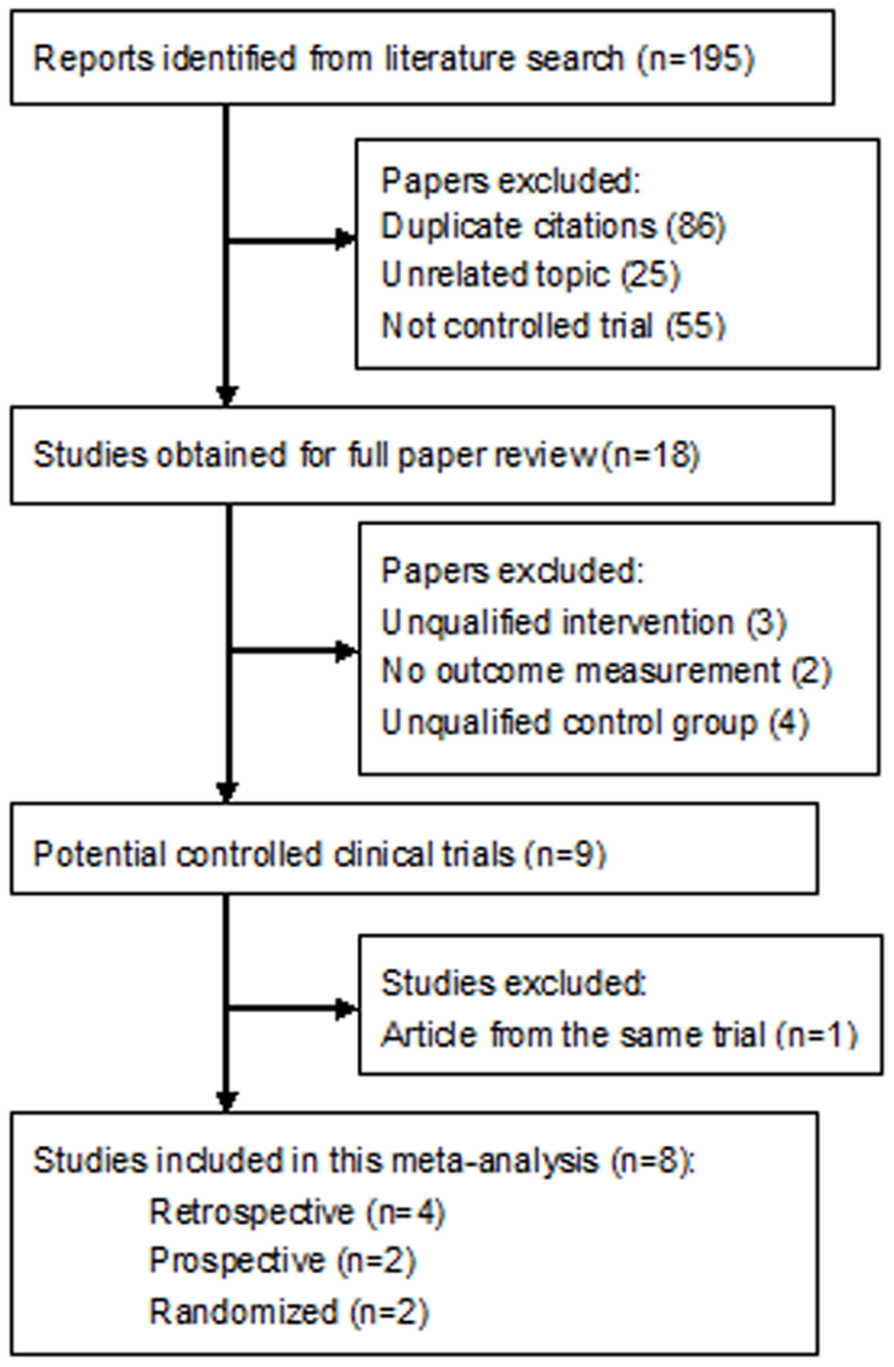
Flow diagram of included studies for this meta-analysis.

A total of 195 articles were initially identified. The abstracts were reviewed and 18 articles with potentially relevant trials were reviewed in their entirety. Subsequently, 9 articles with full texts that met the inclusion criteria were assessed [6], [7], [8], [11], [12], [13], [14], [15], [16]. Two article was from the same clinical trial, and the most recent article was chosen [6], [12]. Hence, a final total of 8 studies published from 2007–2012 were included in this meta-analysis. Figure 1 provides a flow diagram of the search results.

### 2. Characteristics and baseline of the included studies

In total, there were 605 eyes from 559 patients included in this meta-analysis; 281 eyes were included in the Ex-Press group, and 324 eyes were included in the Trab group. The mean age ranged from 62.4–76.35 years. The characteristics of the studies included are summarized in Table 1. The study design was retrospective in 4 studies [8], [11], [14], [15], prospective non-randomized in 2 studies [13], [16], and randomized clinical trials (RCT) in 2 studies [6], [7]. The studies had the following geographic distribution: 4 in the USA, 1 in Spain, 1 in Japan, 1 in the Netherlands, and 1 in South Africa. The mean duration of follow-up ranged from 9.1–65.6 months.

**Table 1:** Characteristics of included studies.

First Author (year)	Design	Location	No. eyes*	No. patients*	Sex(male/female)		Age (year)	Follow-up(mo)
					Ex-Press	Trab		
Maris (2007)	Retro	USA	50/50	49/47	24/26	24/26	66.4/66.5	10.8/11.2
Gallego-Pinazo (2009)	Pro	Spain	20/40	17/20	8/9	9/11	75.0/76.4	9.7/10.3
de Jong (2011)	RCT	Dutch	39/39	39/39	19/20	27/12	62.4/68.6	65.6/66.4
Good (2011)	Retro	USA	35/35	35/35	19/16	17/18	68.9/69.3	28/28
Marzette (2011)	Retro	USA	76/77	69/65	36/40	32/45	66.9/66.8	9.1/9.2
Sugiyama (2011)	Pro	Japan	10/11	10/11	8/2	5/6	64.2/71.3	12/12
Dahan (2012)	RCT	South Africa	15/15	15/15	10/5	10/5	65.4/65.4	23.6/23.6
Seider (2012)	Retro	USA	36/57	36/57	28/8	40/17	71.0/70.8	12/12

* Ex-Press group/Trab group; RCT  =  prospective randomized controlled trial; Retro  =  retrospective; Pro  =  prospective non-randomized; NA  =  not available; Ex-Press  =  Ex-Press implantation; Trab  =  trabeculectomy.

### 3. Quality assessment

The quality assessment is summarized in Table 2. For the Downs and Blacks score, all studies were over 16 (50%), and both the RCTs were 24 (75%). Among all the studies, 6 of 8 trials contained more than 20 eyes in both the Ex-Press and Trab groups.

**Table 2:** Quality scoring components for 8 clinical trials included.

	Quality score component					Score	
First Author (year)	I	II	III	IV	V	Over all	Percentage (%)
Maris (2007)	11	2	5	3	3	24	75.00%
Gallego-Pinazo (2009)	7	2	4	3	1	17	53.13%
de Jong (2011)	11	3	4	3	3	24	75.00%
Good (2011)	9	2	4	2	2	19	59.38%
Marzette (2011)	11	2	4	3	3	23	71.88%
Sugiyama (2011)	11	2	4	3	0	20	62.50%
Dahan (2012)	11	2	5	5	1	24	75.00%
Seider (2012)	11	3	4	2	3	23	71.88%

### 4. Efficacy analysis

Seven studies involving 538 eyes compared Ex-Press with Trab in terms of the IOPR%. No statistical heterogeneity was observed between studies (χ2 = 2.25, P = 0.90, I^2^ = 0.0%). The combined results showed that both surgical procedures significantly decreased the IOP. Ex-Press was found to archive a numerically greater IOPR% from baseline; however, the differences in the IOPR% were not all statistically significant (WMD = 2.33, 95%, CI: −2.59–7.24) (Table 3). We then divided the studies into 3 subgroups according to study design (retrospective, prospective non-randomized, and randomized). All subgroups showed that Ex-Press was associated with a numerically higher IOPR% relative to Trab, but no significant difference was found (Table 4). There was no significant heterogeneity in these analyses. Publication bias was also tested using the Begg test (P = 0.55) and the Egger test (P = 0.53), and both produced non-statistically significant results, indicating a low possibility of publication bias.

**Table 3:** Percentage IOP reduction from baseline comparing Ex-Press implantation with trabeculectomy.

Trial	Ex-Press		Trab		WMD(Random)(95%CI)
	No. Eyes	IOPR% [Mean(SD)]	No. Eyes	IOPR% [Mean(SD)]	
Maris (2007)	50	46.18(45.04)	50	47.06(36.08)	−0.88(−16.88,15.12)
Gallego-Pinazo (2009)	20	43.36(25.31)	40	37.75(16.18)	5.61(−6.56,17.78)
de Jong (2011)	39	51.69(26.27)	39	45.89(29.95)	5.80(−6.70,18.30)
Marzette (2011)	76	50.93(33.79)	77	44.62(36.09)	6.31(−4.77,17.39)
Sugiyama (2011)	10	48.52(14.81)	11	50.33(6.67)	−1.81(−11.80,8.18)
Dahan (2012)	15	44.13(29.35)	15	47.91(43.45)	−3.78(−30.32,22.76)
Seider (2012)	36	35.32(34.83)	57	35.96(34.48)	−0.64(−15.12,13.84)
Total	246		289		2.33(−2.59,7.24)
Test for heterogeneity χ2 = 2.25, df = 6, p = 0.90					
Test for overall effect z = 0.93, p = 0.35					

CI  =  confidence interval; IOP  =  intraocular pressure; IOPR%  =  percentage intraocular pressure reduction; WMD  =  weighted mean difference; Ex-Press  =  Ex-Press implantation; Trab  =  trabeculectomy.

**Table 4:** Sensitivity analysis to evaluate the effect of trial design on percentage IOP reduction.

	Studies (*n*)	WMD(Random) (95%CI)	Heterogeneity			Overall effect	
			*Q*	*P*	*I* ^2^ (%)	*Z*	*P*
All trials	7	2.33(−2.59,7.24)	2.25	0.90	0.00%	0.93	0.35
Retro	3	2.67(−5.04,10.38)	0.80	0.67	0.00%	0.68	0.50
Pro	2	1.18(−6.55,8.90)	0.85	0.36	0.00%	0.30	0.77
RCT	2	4.06(−7.25,15.37)	0.41	0.52	0.00%	0.70	0.48

CI  =  confidence interval; IOP  =  intraocular pressure; RCT  =  prospective randomized controlled trial; Retro  =  retrospective; Pro  =  prospective nonrandomized; WMD  =  weighted mean difference.

Of the 5 studies that reported the probability of complete success, no significant difference was found between Ex-Press and Trab (pooled OR  = 0.93 [0.39,2.23 ]) (Table 5). There was also no significant difference between Ex-Press and Trab in the sensitivity analyses according to study design (pooled OR  = 0.80 [0.27,2.39] of retrospective; OR  = 1.68 [0.68,4.11] of randomized). Six studies reported the proportion of patients achieving target end-point IOP with or without medications at follow-up end-point; the difference in qualified success rate between the Ex-Press group and the Trab group was not statistically significant (pooled OR  = 1.00 [0.39,2.56]). For the subgroup analysis according to design, no statistical heterogeneity was shown between studies, and the difference between groups was not statistically significant (pooled OR  = 1.60 [0.89, 2.89] of retrospective; OR  = 0.26 [0.00, 21.83] of prospective non-randomized; OR  = 0.33 [0.01, 8.22] of randomized) (Table 5).

**Table 5:** Complete success and qualified success comparing Ex-Press implantation and trabeculectomy.

Trial	Studies (*n*)	Success rate, n/N(%)		OR(95%CI)	Heterogeneity			Overall effect	
		Ex-Press	Trab		*Q*	*P*	*I* ^2^ (%)	*Z*	*P*
Complete success									
All trials	5	163/236(69.07%)	176/258(68.22%)	0.93(0.39,2.23)	17.51	0.00	77.20%	0.15	0.88
Retro	4	140/197(71.06%)	158/219(72.15%)	0.80(0.27,2.39)	16.28	0.00	81.60%	0.40	0.69
RCT	1	23/39(58.97%)	18/39(46.15%)	1.68(0.68,4.11)	-	-	-	1.13	0.26
Qualified success									
All trials	6	181/216(83.80%)	212/259(81.85%)	1.00(0.39,2.56)	11.48	0.04	56.40%	0.00	1.00
Retro	3	124/147(84.35%)	132/169(78.11%)	1.60(0.89,2.89)	0.75	0.69	0.00%	1.57	0.12
Pro	2	19/30(63.33%)	41/51(80.39%)	0.26(0.00,21.83)	8.36	0.00	88.00%	0.59	0.55
RCT	1	38/39(97.44%)	39/39(100.00%)	0.33(0.01,8.22)	-	-	-	0.68	0.50

Retro  =  retrospective; Pro  =  prospective nonrandomized; RCT  =  prospective randomized controlled trial; CI  =  confidence interval; OR  =  odds ratio; Ex-Press  =  Ex-Press implantation; Trab  =  trabeculectomy.

### 5. Tolerability analysis

Adverse events in controlled clinical trials comparing Ex-Press and Trab are showed in Table 6. Hypotony and hyphema were two of the most commonly reported postoperative adverse events. Ex-Press was associated with a significantly lower frequency of hypotony and hyphema than Trab, with pooled ORs of 0.29 (0.13, 0.65) and 0.36 (0.13,0.97), respectively. However, no significant differences between Ex-Press and Trab were found with respect to the incidence of choroidal effusion, flat anterior chamber, hypotony maculopathy, bleb leak, or endophthalmitis, with the pooled ORs being 0.65 (0.24,1.80), 1.06 (0.36,3.07), 0.56 (0.16,1.98), 1.41 (0.84,2.39), 1.04 (0.10,10.49), respectively.

**Table 6:** Adverse events from Ex-Press implantation and trabeculectomy compared.

Adverse events	Studies (*n*)	Crude event rate, *n*/N		OR (95%CI)	Heterogeneity			Overall effect	
		Ex-Press	Trab		*Q*	*P*	*I* ^2^ (%)	*Z*	*P*
Hypotony	7	26/246	74/289	0.29(0.13,0.65)	10.47	0.11	42.70%	2.99	0.003
Choroidal effusion	6	24/231	46/274	0.65(0.24,1.80)	11.36	0.045	56.00%	0.83	0.41
Flat anterior chamber	5	9/190	8/192	1.06(0.36,3.07)	2.93	0.57	0.00%	0.1	0.92
Hypotony maculopathy	2	4/126	7/135	0.56(0.16,1.98)	0.05	0.83	0.00%	0.9	0.37
Hyphema	7	4/249	20/271	0.36(0.13,0.97)	2.87	0.83	0.00%	2.02	0.043
Bleb leak	6	38/226	34/249	1.41(0.84,2.39)	2.85	0.72	0.00%	1.29	0.20
Endophthalmitis	2	1/60	1/61	1.04(0.10,10.49)	0.88	0.35	0.00%	0.03	0.97

CI  =  confidence interval; OR  =  odds ratio; Ex-Press  =  Ex-Press implantation; Trab  =  trabeculectomy.

## Discussion

Glaucoma is one of the leading causes of irreversible blindness in the world [1]. Surgical intervention is often needed in glaucoma patients who experience visual field deterioration or progressive optic neuropathy, despite maximum pharmacologic intervention, laser therapy, or both [17]. Trab is the most commonly performed invasive surgical treatment for reducing IOP by creating a communication between the anterior chamber and the subconjunctival space through a guarded sclerectomy. Its success rate and complications are well established [2].

In recent years, Ex-Press has been introduced as an alternative to Trab. Numerous studies have reported on the biocompatibility, safety, and efficacy of Ex-Press during its evolution over the last decade [18], [19], [20], [21]. The device is placed under a partial-thickness scleral ﬂap, and, as is done with Trab, aqueous humor is allowed to collect and drain into a bleb formed in the sub-Tenon space. There are, at present, many available published clinical trials comparing the efficacy of Ex-Press and Trab [5]. However, these trials have usually shown conflicting results, making it difficult to draw conclusions that could be applied in clinical practice. Therefore, the present meta-analysis was undertaken to assess the efficacy and tolerability of both surgical procedures in the treatment of uncontrolled glaucoma.

In the present meta-analysis, we reviewed 8 controlled clinical studies, including respective and prospective non-randomized and randomized studies, using a wide range of clinically relevant outcome measures. In assessing the IOP, our study found that Ex-Press was associated with IOP-lowering efficacy comparable to that of Trab, with a numerically higher but nonsignificant percentage reduction in the IOP. With respect to overall success, both qualified and unqualified, Ex-Press seems to achieve results similar to those of Trab. However, Ex-Press was better tolerated than Trab, with a significantly lower frequency of hypotony and hyphema.

The reasons for this greater tolerance may relate to differences inherent in each procedure. In the traditional Trab, it is difficult to predict filtration volume. As a result, hypotony tends to occur more often. The small internal 50-µm diameter of Ex-Press is unlikely to cause hypotony due to overfiltration [3], [6], [7]. The Ex-Press procedure does not require the creation of an iridectomy, which is commonly performed with Trab, possibly resulting in greater inﬂammation and an increased likelihood of hyphema with the latter procedure [3], [14].

The strengths of the current meta-analysis are as follows. Firstly, the relatively high number of the included studies and cases provide a significant degree of power for the analysis. Secondly, the likelihood of bias was minimized by developing a detailed protocol before initiating the study, by performing a meticulous search for published studies, and by using explicit methods for study selection, data extraction, quality assessment, and statistical analysis. Thirdly, the sensitivity analysis demonstrates that the conclusions from this analysis were robust. In addition, Begg and Egger tests indicated a low possibility of publication biases.

Despite these advantages, the results of our meta-analysis should be interpreted with caution, given certain limitations of the current study. Firstly, we cannot fully exclude publication bias. To avoid publication bias, we conducted not only an electronic search but also a manual search to identify all potentially relevant articles, including published and nonpublished ones. Unfortunately, it is possible that we may have failed to include some papers, especially those published in languages other than English or Chinese. Secondly, although no significant heterogeneity was found, the studies were carried out with small or very small sample sizes, inadequate allocation concealment, and inadequate or no double-blinding. These factors can affect the interpretation of the results. Thirdly, our analyses of the IOPR%, the success rate, and adverse events were based on data pooled from trials of different durations, owing to the lack of data reported in all phases of follow-up. It was a compromise proposal to choose the data from the follow-up end-point. Another potential source of heterogeneity in the results are the assessment criteria of success. Success was defined as target end-point IOP, and there were several different criteria for the normal IOP, such as IOP <18, ≤18, and ≤21 mmHg. Although such assessments of success are widely used as outcome measures in clinical trials, further research is still needed to fully determine their validity, reliability, and sensitivity, so as to ensure that the best is chosen. Finally, some of the controlled clinical trials included in the analysis are not prospective RCTs, but retrospective or prospective non-randomized trials, which may fail to detect actual results.

Ex-Press is considered a relatively new technique compared with Trab. As such, the same surgeon would probably have more experience doing a Trab than an Ex-Press procedure. Therefore, as ophthalmologists become increasingly experienced with Ex-Press, it may produce better outcomes. In addition, even though longer-term results are lacking in this meta-analysis, the study by de Jong et al. [6], which has a mean follow-up period of five years, showed that Ex-Press had better IOP control, fewer antiglaucomatous medications, and a higher success rate compared with Trab. With the increase in availability of more studies with longer follow-up times, there could potentially be a change in the findings of this meta-analysis. Leaving the limitations aside, our findings indicate that at least similar success can be achieved with both Ex-Press and Trab at present.

Taken together, this is the first meta-analysis specifically answering the question of whether Ex-Press is effective and safe for treating glaucoma, on the basis of data available at the present. Even with the limitations, we believe that the results of the current meta-analysis are credible and clinically useful for treatment considerations. Pragmatic randomized controlled trials lasting longer and with broader population inferences are also needed to further confirm our results.
